# Complexity and biosemiotics in evolutionary ecology of zoonotic infectious agents

**DOI:** 10.1111/eva.12503

**Published:** 2017-06-29

**Authors:** Michael Kosoy, Roman Kosoy

**Affiliations:** ^1^ Division of Vector‐Borne Diseases Centers for Disease Control and Prevention Fort Collins CO USA; ^2^ Global Health Asia Mahidol University Bangkok Thailand; ^3^ Department of Genetics and Genomic Sciences Icahn School of Medicine at Mount Sinai New York NY USA

**Keywords:** bacteria, biosemiotics, complexity, disease evolution, host–parasite interactions, infection ecology, pathogen

## Abstract

More is not automatically better. Generation and accumulation of information reflecting the complexity of zoonotic diseases as ecological systems do not necessarily lead to improved interpretation of the obtained information and understanding of these complex systems. The traditional conceptual framework for analysis of diseases ecology is neither designed for, nor adaptable enough, to absorb the mass of diverse sources of relevant information. The multidirectional and multidimensional approaches to analyses form an inevitable part in defining a role of zoonotic pathogens and animal hosts considering the complexity of their inter‐relations. And the more data we have, the more involved the interpretation needs to be. The keyword for defining the roles of microbes as pathogens, animals as hosts, and environmental parameters as infection drivers is “functional importance.” Microbes can act as pathogens toward their host only if/when they recognize the animal organism as the target. The same is true when the host recognizes the microbe as a pathogen rather than harmless symbiont based on the context of its occurrence in that host. Here, we propose conceptual tools developed in the realm of the interdisciplinary sciences of complexity and biosemiotics for extending beyond the currently dominant mindset in ecology and evolution of infectious diseases. We also consider four distinct hierarchical levels of perception guiding how investigators can approach zoonotic agents, as a subject of their research, representing differences in emphasizing particular elements and their relations versus more unified systemic approaches.

## 1 INTRODUCTION

The need for an evolutionary basis in studying and interpreting ecology of infectious diseases, virulence trade‐offs, transmission mechanisms, spatial structuring, competition between microbial strains, and within‐host pathogen dynamics is now well accepted. Examples can include understanding how phenotypic variations of microbes determine the infectious process in hosts, how animals adapt to a long‐term exposure to pathogens, how new mutations or laterally transferred genetic material can lead to increasing virulence or antibiotic resistance in microbial populations, how the size and connectivity of animal populations contribute to pathogen persistence, how a human‐driven transformation of environment can result in emergence of new zoonotic pathogens, and so on (Ewald, 1996; Galvani, 2003; Levin, Lipsitch, & Bonhoeffer, 1999).

The current challenge is the realization that accumulation of information reflecting the complexity of pathogens and infectious diseases has not necessarily lead to major breakthroughs in understanding the evolutionary ecology of infections. The wide application of genetic markers in the study of infectious agents from the 1980s and adaptation of genomic sequencing in the 2000s have resulted in “revolutionary” changes in the laboratory practice. Alas, these technological developments were rarely accompanied by substantive changes in the design of field studies and the conceptual frameworks for analysis and interpretation of the generated data (Achtman & Wagner, 2008). The existing conceptual approaches to infectious disease analyses can still be represented, at their best, by the monumental work on the population biology of infectious diseases by Anderson and May (1979). This theoretical framework preceded the arrival of the massive and complex information produced by genomics, proteomics, and other “omics,” and, unfortunately, is not adaptable to effective integration of the newly available Big Data. The present paper is intended to provoke discussions on this topic. We propose conceptual tools developed in the realm of interdisciplinary sciences of complexity and biosemiotics for extending analyses beyond the currently dominant mindset in ecology and evolution of infectious diseases.

## 2 PATHOGENS AND COMPLEXITY

The classical criteria for defining pathogens, generally known as “Koch's postulates,” have been successfully relied upon for identification of causal relations between microorganisms and pathological manifestations in hosts for over a century. Simply, the criteria state that (i) pathogenic agent has to be present in every case of the disease, but not in organisms without the disease, (ii) pathogen should be cultured from the infected host, (iii) the obtained culture can cause the same disease when inoculated into a healthy animal, and (iv) the pathogen can be isolated from the experimentally infected animal. Although some limitations of these criteria became evident soon after their proposal, for example, inability of culturing some pathogens, the postulates still serve as a guiding light to define a relation between specific microbes and diseases in animals and people. The importance of the Koch's postulates is hard to overestimate; otherwise, how can we claim a connection between a specific microorganism and the disease when many microorganisms are found within a sick animal or a human?

Such strict criteria for defining zoonotic pathogens became challenging following application of modern molecular and genetic techniques, which transformed diagnostics and epidemiology of infectious diseases. High‐throughput sequencing technologies have proved useful for epidemiological surveillance of zoonotic diseases, but without taking into account biological contexts high sensitivity of these powerful tools may also introduce biases and lead to erroneous conclusions (Achtman & Wagner, 2008; Galan et al., 2016). Expanded information on diversity detectable in microbial populations and multiple molecular parameters used for evaluating virulence and immune responses is coincidental with greater uncertainties in defining biological roles of infectious agents as pathogens and their ecological relations with hosts (Kosoy, 2013).

Importantly, when interpreting the results from sequencing and other DNA‐based detection methods, we have to admit at this point that those techniques do not detect microorganisms—they detect particular genes, or, more often, specific fragments of particular genes. Surely, all investigators are aware of this fact, but it is often ignored or is arbitrarily used for interpretation of obtained data. We have to be careful about claiming that the detected nucleic acids can be considered to be the *pathogen*, the term indicating a microbe directly responsible for the damage incurred on the host when reporting a detection of microbial DNA within the host. In the cases of diagnostics based solely on genetic identification, absence of an isolated viable bacterium precludes either experimental verification of pathogenic properties or multisided characterization of the bacterial species. Development of the next‐generation sequencing‐based approaches empowered detection of the so‐called virulence factors, or “pathogenicity islands,” which, again, are also *molecular* and *genomic components*, and not “real” pathogens in terms proposed by Koch and commonly used by others. Such reliance on the identification of a limited number of molecular or genetic markers as a proxy for detection of a biological entity can lead to a critical over‐simplification of the microbial complexity present within a host.

Foreseeing difficulties, the pioneers of molecular diagnostics, Fredericks and Relman (1996), proposed an extension of Koch's postulates to define pathogens relying on genetic evidence alone. These include (i) a DNA sequence belonging to a putative pathogen should be present in most cases of an infectious disease, (ii) the DNA should be found preferentially in the diseased organs, (iii) the DNA should be present only in low amounts or absent in hosts without the disease, (iv) a decrease in the amount of pathogen‐associated DNA should be associated with the resolution of disease, and (v) the nature of the microorganism inferred from the available sequence should be consistent with the known biological characteristics of that group of organisms. The last point should be stressed as especially important as it is commonly ignored by molecular biologists for a simple reason that relevant biological information is often unavailable for most detected genetic factors. An important assumption for the utilization of molecular and genetic approaches is the ability to identify the microbial species, significantly improved by targeting multiple genes/loci (Tibayrenc, 2005). Unfortunately, this reliance on the molecular typing for diagnostic purposes ignores the fact that a biological species, including a microbial species, is a biological category reflecting particular morphology, physiology, behavior, ecology, and evolution; and not just a combination of selectively targeted molecules or genes. It is critical to remember that infections are caused by pathogenic microorganisms or viruses, not by DNA or RNA molecules.

## 3 PATHOGENS AND METABIOTA

Another level of complexity in the investigation of host–pathogen interaction is illustrated by the improved knowledge about the interactions of microorganisms occupying a single animal host organism. Co‐occurrence of multiple infectious agents in the same animal host is very common and can be considered as a rule rather than an exception, supported by a wide application of 16S rRNA amplicon sequencing (Galan et al.,^2016^). Importantly, co‐infection can dramatically change the pathogenicity of specific microbial populations. For example, a recent study (Pagliuca et al., 2016) established that the presence of *Bacteroides fragilis*, an anaerobic bacteria, in the gut microbiota is able to prevent liver damage in mice caused by a vector‐borne bacterium (*Bartonella henselae*). Thus, hosts harboring large number of infectious agents are different from those harboring small numbers and mixed infections can dramatically alter population dynamics of a particular host–pathogen interaction (Cox, 2001). Thomas, Watson, and Valverde‐Garcia (2003) demonstrated that nonvirulent fungi, rarely given attention and largely undetected in the field, can play a significant role in mediating the outcome of coupled host–pathogen interactions in mixed infections. The ability to respond to different environmental factors can play a major role in the coexistence of microbial species demonstrated in experimental communities of *Escherichia coli* B and T‐type bacteriophage, where the trade‐off between resistance to bacteriophage and the ability to compete for the resources resulted in different ecological types of *E. coli* (Bohanan, Kerr, Jessup, Hughes, & Sandvik, 2002).

The infections of the same host by microbe species do not need to occur concurrently to affect the potential outcome of infection by a particular pathogen. An obvious example is pathogens responsible for secondary infections, where an otherwise innocuous microbe can lead to serious infections due to an altered state of the host's immune system. The primary infection can lead to an immunocompromised host as a result of a temporary or long‐term exhaustion of the immune system (HIV and a large number of typically less fatal pathogens, as examples), or, alternatively, through misdirection of the immune response. An example of the latter can be observed by skewing the balance between canonical Th1 and Th2 immune responses, as these are largely exclusive of each other through the cross‐inhibitory actions of IFN‐g and IL‐4 cytokines. Primary infection eliciting Th2 response (such as extracellular parasites) can prevent the host from mounting a Th1 response (typically targeting intracellular pathogens) which normally would adequately control the microbes, allowing for an otherwise benign microbe to behave as a pathogen.

The situation becomes even more complicated when we consider the entire microbiome of a host as a whole system. Microbial communities inhabiting the same animal organism as their ecological niche are involved in the complex interactions through the competitive consumption of energetic resources, cross‐immunity, horizontal exchange of genes, multispecies biofilm formation, and many other modes of environmental manipulation incompletely understood at this time (Fierer et al., 2012; Little, Robinson, Peterson, Raffa, & Handelsman, 2008). Applying ecological concepts to the microbiome, Fierer et al. (2012) considered how microbiome is able to alter host susceptibility to microbial pathogens, aid in the digestion of complex polysaccharides, produce metabolites required by the host, modulate the immune system, regulate environmental conditions within body habitats, and influence tissue development. Host–microbiome interactions span the spectrum from being a symbiont beneficial to the host or having no detectable influence on host health to being a life threatening to the host. Discussing the effect of the microbiome on Crohn's disease, Fischbach and Segre (2016) provided evidences that the gut communities of a subset of Crohn's patients are characterized by a bloom of *E. coli*, and a recent publication by Hoarau et al. (2016) implicates potential contribution to the same disease by a fungal species, *Candida tropicalis*. Another very recent example is the impact of microbiota on the colonization of the mammalian intestine by *Salmonella* (Miki, Goto, Fujimoto, Okada, & Hardt, 2017).

Extending the importance of the host's entire microbiota further, Rogers, Hoffman, Carroll, and Bruce (2013) suggested that the microbiota associated with a particular macro‐organism could represent entities analogous to individual species. Stress was made on importance of both observed and potential horizontal gene exchange between the established pathogens and the bacteria considered to be harmless symbionts. For example, inflammation within the gut can boost gene transfer between pathogenic and commensal Enterobacteriaceae (Stecher et al., 2012), with the commensal bacteria now potentially contributing to a pathogenic effect. Equally importantly, the potential for the transfer of antibiotic resistance, while not changing the definition of pathogenicity per se, greatly affects the available options of responding to the infection, and, thus, affect the ability of pathogenic microbes to spread to new hosts.

The rapid progress in the development of much more sensitive detection methods and the studies of human and animal microbiota, as a combination of all microbes (bacteria, fungi, protozoa, and viruses) inhabiting the same organism, has challenged the criteria for evaluation of pathogenicity status. This trend led also to a greater *terra incognita* separating microbes considered to be a part of “normal” microflora, and those considered to be aberrant and pathogenic (Kosoy, 2013). The increased abundance of data is no longer consistent with the simple model of pathogen versus nonpathogen dichotomy accepted previously as the ever more complex microbiotas reported across a range of chronic infections fit poorly into classical models where simple microbe–outcome associations imply causality (Rogers et al., 2013). Defining the concept of pathobiome, Vayssier‐Taussat et al. (2014) stated (i) an accurate knowledge of the microorganism community defining it, (ii) clear evidence of any effect this microorganism community has on pathogenesis, (iii) an understanding of the impact of the microorganism community on persistence, transmission and evolution of pathogenic agents, and (iv) knowledge of biotic and abiotic factors that may disrupt a stable pathobiome and lead to the onset of pathogenesis. These aspects represent new scientific issues of remarkable complexity and constitute major research challenges in area of infectious pathology.

## 4 HOSTS AND COMPLEXITY

Although the importance of animal hosts in understanding the dynamics and evolution of zoonoses is beyond argument, an evident imbalance between “pathogen‐centered” and “host‐centered” can be observed toward the former. However, microbial properties can be expressed as pathogenic only in the context of a suitable host. A definition of the host of zoonotic agents is itself not as simple as it might first appear because it should specify the role of the animal in the circulation and maintenance of the infection, and “the question of what is a host is very complex, particularly with respect to defining host boundaries” (Casadevall & Pirofski, 2015).

Commonly, a vertebrate animal is called a host for a particular infectious agent isolated from this animal. However, an animal which can carry the microbe for a short time without the ability to maintain it over longer terms could not be an essential host. The practice of screening animal populations by PCR has created an even more sensitive dilemma of treating all DNA‐positive animals as hosts for particular pathogens. Thus, rational interpretation of data obtained during microbiological and molecular surveys from biological and ecological perspectives is required. Estimation of the effect on an individual animal organism may require experimental work, while the effect on the host population may require doing longitudinal field studies. Both ways are time and effort consuming and practically cannot apply in each particular situation.

Another question is about defining a specific category of the animal host. According to Hubalek and Rudolf (2011), the *reservoir host* is a vertebrate species ensuring a long‐term persistence of the agent, the *amplifying host* enables amplification of the agent after initial infection, and the *competent host* is the vertebrate species able not only to amplify the agent, but also to transmit it to another susceptible host or to arthropods serving as vectors or independent reservoirs. The reservoir can be defined not only as a particular animal species, but also as epidemiologically connected populations or environments in which the pathogen can be maintained for a long time (Haydon, Cleaveland, Taylor, & Laurenson, 2002). Identification of the particular functional role of an animal host is a very important and challenging task. For example, among the 203 rodent species or subspecies and 14 lagomorph species reported to be naturally infected with the plague agent, *Yersinia pestis*, only a small fraction of species can be considered to be significant hosts of plague (Gage & Kosoy, 2005). Certain rodent species and their fleas could be considered together as a reservoir host being able to maintain plague in the absence of other rodent species. Epizootic hosts are the species, which routinely become infected, but are incapable of supporting long‐term maintenance of *Y. pestis* in a particular focus although they can be important in spreading the disease during epizootics (Gage & Kosoy, 2005).

Practically, all studies intended to identify reservoir host concentrate on the species level, for example, claiming the role of the deer mouse (*Peromyscus maniculatus*) as a reservoir host of hantaviruses in Northern America. This definition is based on multiple ecological and epidemiological studies, but it is complicated by intraspecies biological diversity as demonstrated by a mitochondrial DNA‐analysis which revealed that animals recognized as *P. maniculatus,* in fact, represent a polyphyletic group (Hogan, Davis, & Greenbaum, 1997). Can all mice of the *P. maniculatus* complex lineages be equally competent reservoir host for Sin Nombre hantavirus? Apart from epidemiological observations and experimental studies, the answer depends on criteria used for demarcation of the rodent lineages and on interpretation of field data. According to a study conducted by Dragoo et al. (2006), phylogeography of deer mice can provide a predictive framework for research on hantaviruses by partitioning mice of *P. maniculatus* into six largely allopatric lineages, some of which may represent unrecognized species. Focusing on lower taxonomic levels, Gómez‐Díaz, Doherty, Duneau, and McCoy (2010) found that detection of *Borrelia burgdorferi* varies greatly among cryptic populations of the *Ixodes* ticks.

In addition to the factors discussed above, many other criteria can be used for identification of functional roles of the animal host: resistance, duration of preservation, level of bacteremia, damage to the organism, transmission competence, occurrence of other microbial species, and so on. The point is that a definition of the role of hosts, similar to the task of defining the role of pathogens, requires *interpretation* of the obtained data. The more data are available, the more thorough and more meaningful interpretation is required.

## 5 DISEASE ECOLOGY AND SCIENCE OF COMPLEXITY

Thus far, most of ecological analyses of zoonotic diseases are based on a duality the host/pathogen relationship, both from the point of the definition of the host and definition of the pathogen. Animals are typically considered as being either infected or noninfected, with some attempts made to evaluate the structure of animal populations for a proportion between “susceptible” and “resistant” individuals. Mathematical modeling of infectious diseases in animal populations routinely use categories of animals such as “infected,” “secondary infected,” “susceptible,” “recovered,” “immune,” and “temporary immune,”. There is nothing wrong with these categorizations, but the vast amount of newly generated data regarding definitions of microbes as pathogens and animals as hosts requires awareness about making *conditioned assumptions* for further definitions.

When we consider the interactions between the parameters presumably essential for these descriptions, the complexity becomes overwhelming. As an example, different scenarios of interference among *B. burgdorferi sensu lato* strains and immune evasion were investigated, revealing that strain interference among pathogens where the presence of one strain affects the fitness of a co‐infecting strain, either through direct competition or indirectly through cross‐immunity (Kurtenbach et al., 2006). This simplified theoretical model was conditionally limited to three host species, one tick species, three genotypes within three *B. burgdorferi* species, an equal abundance of the strains at all times, and mortality rates unaffected by infection with three potential scenarios. In the first scenario, all genotypes of all bacterial species equally evade the innate and acquired immune responses of all host species, and differences among genotypes in the prevalence of infection in larvae feeding on the hosts are innate characteristics of the bacteria and are not immune‐mediated. In the second scenario, all genotypes of a species are equally able to evade the acquired immune response of a host species, but each genotype can only evade the innate immune response of one host species. In the third scenario, the bacteria are successful in evading host acquired immunity for a short period only. The authors illustrated the complexity of this system well, but it is also remarkable how many reasonable assumptions are still to be made.

Here, it is worthwhile to point that complicated is not the same as complex. Many systems are complicated in the way of having numerous moving parts, but what makes a system complex are deviations from simple relationships between components: in complex relationships one plus one does not always equal two. Relating to infectious systems, Casadevall, Fang, and Pirofski (2011) stated “even with complete knowledge of microbes and hosts, the outcome of all possible interactions cannot be predicted for all microbes and all hosts” (p. 2).

There is one more essential dimension is evaluating the complex character of all zoonotic diseases in nature: environmental complexity. Abiotic factors can affect zoonotic agents directly, via changes in host organism, through changes in biotic communities, etc. The concept of a zoonotic system as a self‐regulated ecosystem evolutionary adapted to the natural environment has culminated in the development of idea of “natural focus of disease” in Soviet literature (Korenberg, 2010). The concept received more interest within the framework of medical geography and the spatial epidemiology of zoonoses. The hypothesis closest to the concept of natural focality of diseases might be the one applied for identification of “refugia” as small local areas (0.3% of the region) with particular environmental signatures that support persistence of hantaviruses in the Southwest USA and are not randomly distributed (Glass, Shields, Cai, Yates, & Parmenter, 2007). The research of environmental drivers of distribution and activity of zoonotic agents and their animal reservoirs increasingly relies on huge amounts of information delivered by application of geographic information systems and satellite imagery (Carver et al., 2015; Eisen & Eisen, 2011) which aims to identify factors creating a unique environment that makes a critical contribution to the ecology of a particular zoonotic disease. The caveat is that effects of environmental change may be routinely unpredictable in how these factors enable or restrict pathogen dispersal and activity in animal populations. The overall resilience of the zoonotic system cannot be usually reduced to a linear relation between selected variables (Plowright, Sokolow, Gorman, Daszak, & Foley, 2008).

Of course, the challenges faced in understanding the ecology of zoonotic agents with the arrival of enormous information reflecting the complexity of microbial agents, animal reservoirs, and environmental parameters are not unique. Research of the complex dynamic processes representing various natural and social systems has generated some common trends, conceptually unified under the name of “science of complexity.” Practically, all natural infections fit the criteria proposed for complex systems—they have many interactive agents, are open to contribution from external factors, affected by positive and negative feedbacks, have nonlinear kinetics, and demonstrate evident emergent properties. Simply, an emergent property is an entirely new trait that develops from smaller component traits and emergent property cannot be analyzed solely in terms of the component elements. Systems with long evolutionary history (i.e., pathogen–host relations) allow for the emergent properties in a growing space of otherwise highly unpredictable states (Krakauer et al., 2011). Evolutionary processes lead to mutually intertwined host–pathogen relationships, where investigation of one side of the relationship without paying sufficient attention to the other side will inevitably fail to bring accurate understanding of the relationship.

Attempts to apply the theory and practical means of complexity science for ecology of infectious diseases have been very limited (Arch‐Tirado & Rosado‐Muñoz, 2009; Casadevall et al., 2011; Kosoy, 2013; Krakauer, 2013). To comprehend the novelty of this approach while avoiding a long review, consider a framework for understanding the characteristics of complexity in biology proposed by Dauer and Dauer (2016) based on the compilation of a variety of definitions from other sources: (i) components are diverse and have diverse responses, (ii) functional relationships are nonlinear, continuous, and interactive, (iii) processes are simultaneous, dynamic, and emergent, (iv) manifestations are conditional and irregular, and (v) interpretation allows multiple representations.

The high microbial variability within most populations of known pathogens is well documented (Achtman & Wagner, 2008; Gupta & Maiden, 2001; Kosoy, 2013). In reality, studies of infectious diseases, whether an experimental animal study or a diagnostic detection of a pathogen, typically focus on a specific type or a species of zoonotic agent. For example, to characterize properties of microbial species or strains investigators are prompted to select either a type strain or specific strains a priori different from the type strain used for the experimental infection. This is understandable, but requires recognition that such experimental approaches are based on the frequently erroneous assumption that all strains belonging to one type (species) are similar. This applies to studies of animals both in vivarium and in the field—the expectation is that the response to the infectious agent will be more or less similar under the same conditions while we realize that we cannot control all of these conditions and that microbial variation should not be ignored.

Nonlinear relationships between components of a zoonotic system mean that the interactions between them are not proportional. For example, the studies of cotton rats of different age groups suggest that prevalence of the infection declined from juveniles and young subadults to adults and old adults (Kosoy, Mandel, Green, Marston, & Childs, 2004). While making such a conclusion we suppose that infectability of rodents changes gradually from a very young age to their death; although in reality, it is quite possibly that the probability of being infected instead has a bimodal distribution, with a combination of factors determining where a particular individual animal fits: reproductive status, past infection history, population density, food availability, and so on, with interactions between each component also likely to be complex.

As discussed earlier, the assumption of simple causality in zoonotic diseases is not well warranted either. The belief in a simple cause is still strong, for example, that a clinical symptom is caused by the presence of a virulent strain or associated with specific variations within a genome. A frequent error made by most researchers is interpreting association as causality. In reality, the observed manifestations can result from many factors and require understanding of the entire zoonotic system to elucidate the significance of each component. When interpreting relationships between infectious agents and their hosts, each situation can be represented in quite different ways depending on the context of natural and social situations. A tricky, but very important situation when analyzing a zoonotic system, is the coordination of complexities observed at different levels of organization: at molecular, cellular, organismal, population, and ecosystem levels. Upon the realization of complexity of both microbes and hosts at each level, the bottom‐up and top‐down interactions between elements at different levels of organization cannot be expected to be simple, linear, and proportional. In complex adaptive systems, such as infectious agents and their hosts, it is rather difficult to measure causality with many interacting variables often with feedback loops. These dynamic systems still manifest causality, but evaluation of a causal contribution of each factor within a complex system is rather challenging (Krakauer, 2013; Krakauer et al., 2011).

## 6 A “SIMPLICITY–COMPLEXITY” CONTINUUM AND SEMIOTIC MEANING

Advances in computational methodology have greatly facilitated handling of the Big Data, whether from molecular biology or from remote sensing, but the question of the biological role of microbes, as zoonotic agents, or animal, as zoonotic hosts, becomes even less certain with the emphasis on the Big Data. Analyses of numerous variables via methods with sophisticated names related to either the microbe or animal host can be perfectly appropriate for building mathematical models to be presented at a meeting, but provide less utility when a clear and convincing definition is required for making urgent and critical decisions: Which pathogen is responsible for the newly emerged outbreak, what range of variability within the infectious agents can we accept for this definition, whether a specific animal species can be defined as a reservoir host, vector host, effective spreader, or simply as an incidental carrier? Between the extremely complex reality and the desired simple explanation, there can exist a wide spectrum of intermediate positions representing a continuum between the entirety of potential probabilities for expression of all components of the infectious system and the simplicity of essential parameters attributed to microbial agents, animal hosts, and specific landscapes with the specific roles they play (Kosoy, 2013).

The main questions for defining the role of microbes as infectious agents, animals as host, or environmental parameters as infection drivers could be phrased in the terms of *meaning* or *functional importance* of multiple variables. What does it mean for a specific microorganism to be considered as a pathogen under specific conditions? Critical parameters may include a known level of diversity within this species, anticipated presence of other microorganisms, estimated density and population structure of specific animals, presence of particular ectoparasites, and potential for survival of the microbes in other arthropods or in soil? These are picked almost randomly; they may have more or less sense (“meaning”) depending on the specific disease and context that dictate significance (*value*) of each parameter. The caveat is that most needed indicators are almost subjective (according to each situation) and their “value” has to *be chosen*.

Talking about *meaning*, we enter the scientific discipline of *semiotics*, and, in particular, *biosemiotics*. During the last couple decades, the science of “Biosemiotics” attracted quite a few experimental and theoretical biologists with regular international conferences and numerous publications, but very marginally reached the area of evolution and ecology of infectious diseases thus far. To the descriptions of biological relationships biosemiotics introduces such unanalyzed concepts as “function,” “information,” “code,” and “signal,” and the use of such terms “points to the fact that those notions cannot be avoided or fully substituted with merely chemical accounts” (Kull, Deacon, Emmeche, Hoffmeyer, & Stjernfelt, 2009, page 170).

Biosemiotics, as a discipline, should not be confused with epistemology. While the epistemology focuses on the comprehension of the relationship between entities, or between an observer and an entity, semiotics deals with a narrower idea of meaning, or sign, as pertaining to such entities. In the latter, we do not need to know all the nuances about the potential relationship, but we can elucidate how one entity can recognize another entity. A question commonly asked from the biosemiotic perspective can be formulated as how can participants of infectious systems in natural settings *recognize* each other. Specifically, how can an infectious agent recognize an animal organism as a “reservoir host” or as “incidental host”? How can a potential host recognize a microorganism as a harmless or beneficial symbiont, or as a potentially damaging pathogen? To which *signs* associated with bacteria does an animal organism refer to when switching its status from “resistant” to “susceptible” in relation to specific pathogens? (Kosoy, 2013). We have to keep in mind that these questions are principally different from the questions of which molecular or cellular mechanisms enable such processes to happen. The list of specific molecular interactions cannot replace a meaning of specific “signs” expressed by microbes to animals, and by animals to microbes, through the two‐ and multiple‐way communicative process. The eventual recognition of the bacteria by the animal organism either as a pathogen or as a part of symbiotic microbial community can be expressed as a definition of them as alien cells (“non‐self”) versus “own” cells (“self”). In a related reference, Turovski (2001, p. 412) wrote: “viewing the parasite‐host relationships as dialogical developments … the most powerful instrument of those dialogical traits appears to be the skillful manipulation of the criteria of ‘own‐strange‐alien’, most important in the intra as well as interspecific relations of both: parasites as well as hosts”.

According to Langwig et al. (2015), the pathogen invasion process is divided into four distinct stages—”pre‐arrival” (pathogen arrival is not imminent), “invasion front” (pathogen invasion has just occurred and host population is stable or initial declines), “epidemic” (pathogen prevalence moderate to high substantially impacting the host population), and “established” (pathogen prevalence is variable but stable and host population is stable). From this point, a mutual recognition of hosts and pathogens will differ at each stage and require differential description of roles that they “play” in their interaction. A recognition of both a pathogen by a host or a host by a pathogen is actually effective only in the specific context. If the de‐contextualization intends to eliminate any contextual or linguistic aspect declared for handling big datasets (Leonelli, 2014), the semiotic approach, in contrast, emphasizes the meaning of the pathogen–host relationships making sense only in the specific context. Similarly, a perception of the pathogen and the host by an investigator also greatly depends on a number of social and cultural factors, including tendencies prevalent in the scientific community affecting not only the description of zoonotic systems, but also decision‐making steps in disease control management.

## 7 PERCEIVED LEVELS OF ORGANIZATION

From an epistemological perspective, the growth of information about diversity of microorganisms is accompanied by an increased awareness about “what we do not know” versus “what we do not know about what we do not know” through the realization of how small is the proportion of “known” data versus “unknown–hidden–invisible” data. The determination of microbes as pathogens becomes ever more problematic, and the same trend can be easily observed with the realization of how the status of the hosts is sensitive to numerous factors and that accumulation of additional data may not significantly contribute to answering the question “what is the host?” Overall, we have to admit the disconnect between the quickly accumulating information on zoonotic agents, hosts, and ecological variables, and our still limited understanding of zoonotic processes and ability to predict them, relating to the question of handling unknown information (Kosoy, 2013).

It is evident that zoonotic pathogens and their relationships with animal host need to be studied at different hierarchical levels of biological organization—molecular, cellular, organismal, population, and ecosystem levels. But there is another kind of hierarchy that we should consider if we accept a necessity of the *meaningfulness* of obtained information. Simply put, a level of perception indicates how investigators approach zoonoses as a subject of their research. Surely, this can represent a level of biological organization, for example, molecular versus population, but can also reflect hierarchical dimensions representing differences in emphasizing the role of individual elements (fragments) versus more systemic approaches.

**Figure 1:**
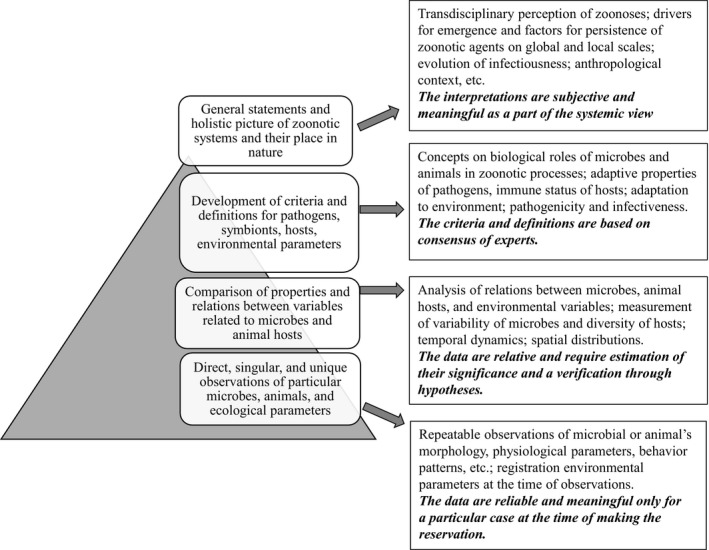
The spectrum of descriptions of zoonotic infectious diseases represented by hierarchical perceived levels. Graphical image of the pyramid behind the descriptions signifies the required incorporation of the lower level perception prior to be able to generate any valid conclusions at the higher perception level. The wider base of the pyramid indicates that support for a higher level of perception must come from a broad range of the underlying observations, indicating the increased inter‐ and trans‐disciplinarity at the higher perception levels

Below are proposed four different levels of perception with some examples and general description for each level (Figure 1). The first level represents immediate (singular, unique) observations with a description of elements of zoonotic systems; for example, a measurement of shape and size of bacteria observed in a microscope, an antibody titer in an animal serum against a specific antigen, PCR amplification of a DNA fragment mapped to a pathogen, observation of specific clinical manifestations, specific reaction of experimental animal to inoculation with known microbial dose, etc. We expect these to be well‐defined, reliable, concrete results, but applicable for a concrete and unique case with observations made under specific conditions while following a strict protocol.

At the second level, the emphasis is made on correlative relationships between individual parameters of microbes, animal hosts, and natural and social environment. At this level of perception, parameters are compared between themselves; for example, comparing sensitivity of animal populations to the infectious agent, detecting temporal changes in humoral immune response, or testing for correlation between ecological factors and infection prevalence. This is still very much a data‐driven approach; however, it may require extrapolating the observed results to sets of comparable organisms, or situations, using appropriate statistical methods. For analyses of observed differences, a demarcation line between compared classes or types is needed and we focus on categories, such as a microbial species defined by a name. When a scientific name is not available, the groups are labeled as types or classes for the purpose of assigning observed parameters to these groups. Based on the observations, hypotheses can be proposed and tested. The results and verification of the hypotheses can be presented as relative and based on the probabilities of occurrences. In other words, this is the level, where we proceed beyond the immediate observation and applying generalization and categorization to extend our understanding within a specific discipline (e.g., microbiology).

At the third level of perception, a primacy of correlation‐focused statistical approaches between separated parameters is replaced by attempts to reveal causal explanation. This is an important step which allows to extend from a simple aggregation of data and observations into assigning the functional notation to the observation as to their contribution to the ongoing process. Here, some conceptual models are proposed; for example, a specific animal species with the capacity to maintain a circulation of the particular pathogen and linked to a suitable transmission vector is considered to be a natural reservoir of the said pathogen. Note the difference—at the first level, we detect a specific bacterium or a virus in the animal; at the second level, we hypothesize that this animal species is more likely responsible for carrying the pathogen; and at the third level, we claim that this animal species is, in fact, the reservoir and therefore, theoretically, reduction of the disease can be achieved by controlling the population of these animals. It is this level of concepts that intends to explain the origins, development, and complexity of pathogens enabling us to make practical decisions. Contribution from different disciplines (e.g., molecular biology, microbiology, immunology, and ecology) effectively requires interdisciplinary approach at the third level.

At the fourth level, the evolutionary history of pathogen–host relations, microorganisms as an inevitable part of animal or human body, the role of pathogens in the regulation of animal populations, and a connectivity between numerous natural and social factors can be considered. As we have a large body of evidence from the previous levels of perception, we can now organize them in such a way as to be able to ask for larger patterns which may explain the observations and identify the commonalities and exceptions, for these observations. Both ethical and practical questions also appear at this level as well, such as whether elimination of animals considered as a source of human diseases, like the culling civets in Asia at the height of SARS outbreaks, is acceptable considering the dramatic changes occurring to the local ecosystem. Here, we can consider whether the decision to control an identified host will in fact reach the ultimate goal of effective control of the developing outbreak, or whether the local availability of other potential hosts or vectors can simply replace the one we chose to target, allowing the epidemics to continue unabated. Overall, this level is correspondent to transdisciplinarity that defines a research strategy that crosses many disciplines to develop a more holistic approach (Nicolescu, 2010). There are some recent successful applications of these approaches for analysis of complexity of zoonotic diseases, in particular, Hendra virus, Nipah virus, avian flu, liver fluke, and cholangiocarcinoma (Plowright et al., 2008; Wilcox & Echaubard, 2016; Ziegler et al., 2016).

To illustrate further the proposed levels of description, let us look at the questions raised for investigation of plague caused by *Y. pestis*. The main questions that have dominated research on plague ecology over last 100 years can be formulated as follows: How can plague persist in specific ecosystems and what is a driving force for emergence of plague in ecosystems after a long absence? We can systematize a diversity of proposed hypotheses by referring them to different levels of perception (Kosoy, 2016). At the first level, the investigations concentrate on particular characteristics of components of the system (phenotypic features and genetic elements of *Y. pestis*, presence/absence of specific species of rodents and fleas, isolation of the bacterium, presence of antibodies, etc.). At the second level, the investigations focus on comparing properties of different components and relations between them (virulence of strains, frequency of mutations, antibiotic sensitivity, antibody level, animal survival time, landscape features, climatic factors, etc.). The third level reflects a development of specific concepts (genomic evolution of *Y. pestis*, L‐form transformation, adaptation of bacteria to specific hosts, transmission mechanism, asymptomatic carrying, survival of the pathogen in soil, metapopulational structure of host population, etc.). Finally, at the fourth level, plague is considered as a self‐regulated ecological system adapted to its environment. Although the hypotheses proposed at the different levels are often considered as competitive and even mutually exclusive, in reality, most of them can work in a synergistic manner and be instrumental for defining a potential framework leading to a more integrated picture of plague as a natural phenomenon (Kosoy, 2016; ).

The descriptions of these levels are unavoidably brief due to the scope and limited size of the present paper, but the main point is that a meaningful interpretation of complexities is specific for each level of perception, and cannot be automatically transferred to another level. Such attempts, called “category error” by Ken Wilber (1996), can cause big confusions and represents a major hazard in describing the real complexity of zoonotic systems and keeping the balance in interpreting this complexity and ability to derive appropriate conclusions.

## 8 LOGIC IN RESEARCH OF ZOONOTIC AGENTS

The generation of massive information during investigation of pathogens and infectious diseases demands meaningful interpretation of the information and its functional capacity. The deep problem lies in our ability to think—to apply logic for producing knowledge rather than just accumulating data and information. Modern science with all its progress is based on binary logic, in other words on the dichotomy of logical constructions, often called traditional or Aristotelian. One of the classical illustrations of this logic is Hamlet's “to be OR not to be”. The description of epidemic systems is still mostly based on binary logic with limited and fixed opposing statuses, for example, “pathogenic microorganism” versus “nonpathogenic”; “infected organism” versus “noninfected”; “resistant organism” versus “susceptible to the infection”, “epidemic” versus “sporadic cases of disease” (Kosoy, 2013). This logic limits the realization and handling of the real complexity and simplifies the hierarchical structure of epidemic systems. Handling the complexity of epidemics at different levels of perception, which assumes different degrees of acknowledgment of our knowledge, requires a new kind of logic –”paradoxical logic” which allows questions such as “how to be AND not to be”, depending on the context, priorities, functional importance, and finally depending on the choice of investigators. As F. Scott Fitzgerald originally wrote in 1936, “the test of a first‐rate intelligence is the ability to hold two opposed ideas in mind at the same time and still retain the ability to function”(Fitzgerald, 2009).

A choice of the perspectives depends mostly on the natural and social contexts, as well on priorities faced by investigators. This choice is based not exclusively on a theoretical foundation, but also on practical experience, knowledge of other scientific disciplines outside our own, philosophical education, intuition, and scientific soundness. A relationship between subjective and objective knowledge in general is not a purpose of our discussion; the claim is that a choice of the perception should reflect expected results and their interpretation. If we talk about “virulence” and use the words “virulent factors” for a description of specific molecules produced by pathogenic bacteria, we have to be aware that criteria for accepting the phenomenon of virulence are not just different, but also could not be appropriate for evaluation of, for example, colonization of a niche in an animal organism, coordination of relationships with coexisting microbes, or evasion of the host immune response. This is just a molecular structure that may or may not be important in causing the pathological manifestation depending on the context. Under some conditions, the presence of such factors can be a critical determination for the progression of zoonotic disease (i.e., emergence of antibiotic resistance in cases of reliance on this antibiotic for disease control), or under other conditions it may be of no consequence whatsoever (i.e., when this antibiotic is not utilized at all). Following the nontraditional logic, it would be more correct to say that this structure may AND may not cause pathology because of both different potential outcomes.

## 9 CONCLUDING REMARKS

There is a common belief that new computational tools can simply channel the “avalanche” of Big Data collected from ecological, genetic, immunological, and microbiological investigations into reasonable conclusions without adjusting our perception of the functional importance of and hierarchical relations between the available variables. This does not imply that the existing approaches for describing and analyzing relations between pathogenic agents and hosts, or between infectious processes and environment are wrong. Instead, the dynamic complexity of zoonotic pathogens revealed by the novel genetic and genomic data, along with extensive environmental parameters involved, requires acknowledgment of the limitations of current approaches, and leads us to propose new ways for interpretation of the data. The main premise of this article is that we need to be flexible in studying natural systems of zoonotic pathogens with respect to how we choose perspectives within a continuum between unrestricted diversity of related parameters and well‐defined roles played by infectious agents, potential and actual animal hosts, and environmental variables. The proposed model of investigation requires a dynamic switch of perspectives along the *simplicity–complexity* (“simplexity”) dimension from virulence factors to multisided descriptions of the pathogens, from individual microbes to ecosystem‐wide microbial communities, from specific clinical manifestations to infectious patterns, from findings of infectious agents to defining a natural focus of the infection as a self‐regulated system, from single factors affecting host–parasite relations to the complex ecological context, and more. A choice of making a meaningful perspective and following interpretations of information based on its functional importance are representations of both the subjective nature of investigations of zoonotic pathogens and much more objectively derived information, for example, coded in the genetic structure of DNA or in observing the morphology or behavior of bacteria.
